# Psychiatry

**Published:** 1904-06-18

**Authors:** 


### Progress in Medicine and Surgery.

### PSYCHIATRY.

*(Concluded from page 189.)*

**Prodromata of the Psychoses.**—Clouston of Edinburgh draws attention[5] to this very important matter. It is a fact, says he, by no means sufficiently recognised, that attacks of mental disease have early symptoms that are often not mental in character, and that all sorts of sensory, vaso-motor, and motor symptoms "may be the mere preludes to an attack of insanity and not of themselves the real disease." Such prodromata are well worthy of study because the recognition of their true character might enable us in many cases to anticipate and possibly to ward off the mental attack. The neurologist who is called in to see a woman suffering from an unusual form of headache with anorexia, insomnia, and obscure paræsthetic sensations, often misses the real point of the case because he does not realise that such symptoms are, in this particular patient, of higher cortical origin, and may mean an attack of acute mania in a week if nothing can be done to arrest their course. Speaking generally, disturbances of common sensibility are by far the most frequent and least serious form of neurosis to which nervous humanity is subject. Many women are seldom without a headache during some part of every 24 hours. In a still greater number headaches are set up by slight causes—indigestion, want of sleep, changes of air and of temperature, worry, menstruation, "excitement" of any kind and alcohol. The case is recorded of a woman, aged 50 years, who had been subject at times to headache and "weary painful feelings" in the back of the neck when tired, but who otherwise was healthy and strong. On the occasion of some domestic trouble she had to do much nursing which aggravated her symptoms. She then had to take to bed to recruit her strength, but she suffered from insomnia and became mentally depressed. When the melancholia came on the bodily pain went off. In the course of three months she had to be placed in a mental hospital for treatment. She recovered, but the first symptom of the passing off of the psychosis was the recurrence of the headache and bodily pains. She has had several such attacks since, each one with the same sequence of symptoms. "She has the dread and impending feeling of danger which so many melancholiacs have before an attack." A certain amount of morbid mental heredity was found in her case. In most cases the headaches which precede mental disorder have not quite the character of the ordinary "woman's headache." They are more constant, more intense, more distracting and disabling, and often accompanied with paraesthesiæ, such as feelings of weight, of lightness in the head, or of a "congested" and "bursting" character. They are seldom localised and are not attended with sickness or symptoms of migraine. They are most frequently and characteristically seen as the prodromata of post-influenzal depressive insanity. The sequence of symptoms is as follows in the case of an oncoming mental disorder:—Sensory disturbances arise first, disturbing impressions are sent to the higher centres (psychical centres), and cause reactions of depression, of excitement, or of stupor. Insomnia sets in early, and loss of appetite and neglect of habits of delicacy are manifested. A supersensitiveness to light and noises is often exhibited, and actual hallucinations of sight, sound, or smell are developed. That general condition of motor instability, known as "fidgets," and muscular unsettledness is exceedingly common. The muscles of facial expression lose their delicacy and subtlety of action, there is a change in the expression of face and eye before the mind gives way. The eye is dull and listless, and the cornea has the glare of acute insanity, the brow is furrowed, and fixed "lines of care" show the oncoming of melancholia. There is often an "abstracted" look on the face, and the play of features of health, which gives beauty and interest to the face, is lost. It is common in the female sex to meet with symptoms of hysteria before the advent of insanity. Circulatory and vaso-motor disturbances, fainting fits, "sinking at the heart," and the "flushings and heats" so common at the menopause, are also among the prodromata of insanity. Constipation and digestive disorders occur in over 50 per cent. of cases as prodromata of various forms—especially the depressive forms—of insanity, while amenorrhœa is a frequent accompaniment. Toxic symptoms arising from auto-intoxication produced by absorption of toxins from the disordered alimentary canal play a part in aggravating the cerebral disturbance, and in producing hallucinatory and acute mental confusion. Early aperient treatment of such cases with calomel and salol will partly relieve such cerebral disturbances. I believe, says Dr. Clouston, that many attacks of insanity are warded off by this means and by appropriate dieting, just as attacks of epilepsy are often so prevented. The acuter insanities and general paralysis are especially apt to be preceded by marked intestinal or gastric catarrh. Loss of appetite, defective digestion and assimilation of food, and loss of body weight may precede an attack of insanity. Dr. Clouston mentions a case where a patient lost six stone in weight in the six months preceding an attack of insanity. It is difficult, if not impossible, to distinguish between prodromata which are not an integral part of the oncoming psychosis, and the early symptoms which form an essential and as it were a fatalistic part of the psychosis itself. Only experience of individual cases can determine this, and it is probable that while the two series of phenomena are distinct in some cases they may gradually blend together in other cases.

[5] Jour. of Mental Sci., April 1904.

